# Scabies in remote Aboriginal and Torres Strait Islander populations in Australia: A narrative review

**DOI:** 10.1371/journal.pntd.0009751

**Published:** 2021-09-30

**Authors:** Prudence Gramp, Dallas Gramp

**Affiliations:** Gold Coast University Hospital, Southport, Queensland, Australia; London School of Hygiene and Tropical Medicine, UNITED KINGDOM

## Abstract

Scabies has recently gained international attention, with the World Health Organization (WHO) recognizing it as a neglected tropical disease. The International Alliance for the Control of Scabies recently formed as a partnership of more than 15 different countries, with an aim to lead a consistent and collaborative approach to preventing and controlling scabies globally. Scabies is most prevalent in low-resource and low socioeconomic areas that experience overcrowding and has a particularly high prevalence in children, with an estimated 5% to 10% in endemic countries. Scabies is widespread in remote Aboriginal and Torres Strait Islander communities in Australia with the prevalence of scabies in Aboriginal and Torres Strait Islander children in remote communities estimated to be as high as 33%, making it the region with the third highest prevalence in the world. This population group also have very high rates of secondary complications of scabies such as impetigo, poststreptococcal glomerulonephritis (PSGN), and rheumatic heart disease (RHD). This article is a narrative review of scabies in remote Aboriginal and Torres Strait Islander populations in Australia, including clinical manifestations of disease and current treatment options and guidelines. We discuss traditional approaches to prevention and control as well as suggestions for future interventions including revising Australian treatment guidelines to widen the use of oral ivermectin in high-risk groups or as a first-line treatment.

Scabies has recently gained international attention, with the World Health Organization (WHO) recognizing it as a neglected tropical disease [1]. The International Alliance for the Control of Scabies recently formed as a partnership of more than 15 different countries, with an aim to lead a consistent and collaborative approach to preventing and controlling scabies globally [2]. In Australia, 10 million dollars was awarded to the Murdoch Children’s Research Institute to implement the World Scabies Elimination Program—an initiative aimed at collecting data from many affected countries and scaling up mass drug administration (MDA) [3].

Scabies is most prevalent in low-resource and low socioeconomic areas that experience overcrowding and has a particularly high prevalence in children, with an estimated 5% to 10% in endemic countries [4,5]. The 2015 Global Burden of Disease Study ranked scabies with the 101st highest disability-adjusted life years (DALYs) estimate out of 246 conditions [6]. This is, however, likely underestimated as secondary complications, such as impetigo and kidney damage, were not included in this study [6,7]. A study from Fiji showed that 94% of impetigo was attributable to scabies [8], and it is estimated that approximately half of the instances of acute poststreptococcal glomerulonephritis (PSGN) in tropical regions can be attributed to skin infections [9].

## Burden of disease in remote Aboriginal and Torres Strait Islander communities

Scabies is widespread in remote Aboriginal and Torres Strait Islander communities primarily as a result of overcrowded housing, poor living conditions, poor access to services, normalization of skin conditions, and high prevalence in younger age groups [10,11]. The prevalence of scabies in Aboriginal and Torres Strait Islander children in remote communities is estimated to be as high as 33%, making it the region with the third highest prevalence in the world [11]. The DALY for Australasia in 2015 was calculated by the Global Burden of Disease Study at 5.22 per 100,000 people, which has increased from 4.97 in 1990 [6]. This does, however, include the whole of Australia and New Zealand, and the DALY for Aboriginal and Torres Strait Islander Australian communities would be significantly higher due to a high prevalence rate.

Aboriginal and Torres Strait Islander children in remote communities also have the highest prevalence of impetigo in the world at 45%, and skin infections and infestations are some of the most common presentations for children attending primary health care centers [11,12]. The Healthy Skin Project in East Arnhem Land found that before children turned 1, they presented on average 3 times for scabies [11]. A study carried out between 1996 and 2012 found that Aboriginal and Torres Strait Islander children born in Western Australia in this time were hospitalized for skin conditions 15 times more than non-Aboriginal children, with 15% of these for scabies [11]. Skin infections with *Streptococcus pyogenes* (Strep A) can lead to PSGN or rheumatic heart disease (RHD), which is disproportionate in Aboriginal and Torres Strait Islander peoples who accounted for 89% of new RHD diagnoses between 2015 and 2017 in Australia, with 75% of these in younger age groups <25 years [7].

## Clinical disease

Scabies most commonly presents with an intensely itchy, erythematous, and papular rash and visible burrows. 4 to 6 weeks after initial contact with an infected host, the patient develops a hypersensitivity skin reaction to the burrowing of the female mite and subsequent laying of her eggs [4]. The patient may have papules, gray linear, or irregular tracks/burrows, which most commonly appear in the web spaces of the hand, palms, wrists, the armpits, and the groin. Diagnosis can be made clinically or by dermatoscopy, light microscopy of skin scrapings, or biopsy [4]. People with darker skin types may be more difficult to diagnose due to lack of erythema and difficulty in identifying dark colored mites on dermoscopy. In 2020, the International Alliance for the Control of Scabies published a guideline for the diagnosis of scabies, including 3 tiers: “Confirmed scabies (level A) requires direct visualization of the mite or its products. Clinical scabies (level B) and suspected scabies (level C) rely on clinical assessment of signs and symptoms” [13]. This guideline aims to standardize scabies diagnosis to improve research and epidemiological studies and support development of future treatment programs [13].

Scabies is very itchy and causes night time scratching that can result in insomnia. In the case of children, this can negatively impact their performance at school. There is significant risk of secondary infections such as Staphylococcal and Streptococcal impetigo or cellulitis, which can lead to sepsis and is potentially fatal [4]. Secondary infection may also lead to rheumatic fever or PSGN, and, therefore, chronic effects on the kidneys and heart [4]. Patients who are immunosuppressed, malnourished, or have neurological or sensory deficits may develop extensive infestations with millions of mites leading to a particularly contagious and significant skin disease called crusted scabies. These patients should be screened for immunosuppression such as HIV/AIDS coinfection [4,10]. Scabies can present differently in remote Aboriginal and Torres Strait Islander communities where rashes on the head and neck are more likely, and there is a higher risk for crusted scabies than in nonindigenous Australians [14].

## Treatment

Scabies is treated with topical or oral scabicides. The first-line treatment in Australia is 5% permethrin cream that is applied to the skin from the neck down and left for a minimum of 8 hours. Oral ivermectin of 200 mcg/kg is only subsided under the Australian Government Pharmaceutical Benefits Scheme as a second-line agent once topical treatment has failed or in the case of crusted scabies [14,15]. It is simple to administer but is slightly less efficacious and more expensive than topical permethrin [4,10]. In Australia, oral ivermectin can be used in children >5 years old and >15 kg and nonpregnant adults [14]. Permethrin 5% cream has been shown to be more effective in treating scabies than oral ivermectin in trial data; however, this may not be applicable to all populations due to external validity issues such as compliance and tolerance [16]. Treatments for infants remain limited in Australia, with the therapeutic guidelines recommending 10% sulfur in white soft paraffin in infants 4 to 6 months and 5% sulfur in white soft paraffin in infants <2 months [14]. Treatments need to be repeated after 7 to 14 days to ensure proper treatment of newly hatched mites [4,10]. If skin infection is present, it needs to be treated concurrently; otherwise, scabies treatment is unlikely to be tolerated [10,17]. In endemic regions such as remote Aboriginal and Torres Strait Islander communities, impetigo is more likely to be due to *Streptococcus pyogenes*, and, therefore, oral or intramuscular antibiotics are recommended instead of first-line mupirocin best suited in other populations [18]. It is recommended to use either 1 dose of intramuscular benzylpenicillin or 3 to 5 days of trimethoprim and sulfamethoxazole [18].

Treatment for pregnant women remains limited. Oral ivermectin is classed as category B3 by the Australian Therapeutic Goods Administration, which means there is limited human data from trials to ensure safety, and observed fetal malformations in animals have unclear significance to human populations [19]. Permethrin 5% cream is recommended as the treatment in Australia for pregnant women and is categorized as B2 with limited human data; however, no fetal malformation in humans or animals have been observed [14,19]. In a small number of countries, such as France, oral ivermectin has been used in pregnant women with no demonstrated fetal adverse effects [20]. Additional management approaches for scabies include identifying and treating close contacts, washing linen, household cleaning, and treating for complications of scabies such as itch or dermatitis [4].

## Prevention and control in remote Aboriginal and Torres Strait Islander communities

Standard approaches to scabies prevention and control have been identifying and treating cases early and isolating and aggressively treating crusted scabies due to its highly contagious nature and risk of significant outbreaks [10]. MDA, usually with oral ivermectin, is seen to be an easy and successful method to prevent and control outbreaks. Environmental decontamination through laundering linen and household cleaning is recommended in many guidelines, although the evidence for the efficacy of this is mixed [10]. In outbreak areas in remote Aboriginal and Torres Strait Islander communities, addressing socioeconomic issues such as poverty, overcrowding, inadequate housing, and poor hygiene practices would contribute to prevention and control of scabies [10].

The main method of control in remote Aboriginal and Torres Strait Islander Australian communities has been education and awareness campaigns coupled with MDA. The National Healthy Skin Guideline: for the Prevention, Treatment and Public Health Control of Impetigo, Scabies, Crusted Scabies and Tinea for Indigenous Populations and Communities in Australia, released in 2018, has developed evidence-based guidelines and identified gaps in knowledge to direct future research [21].

Following a whole of community-based treatment program in Panama, a similar program was trialed in some remote Aboriginal and Torres Strait Islander communities in the Northern Territory in the late 1990s with good success, reducing scabies prevalence from 32% to 10% [12]. The challenge that has been identified, however, is maintaining low infestation rates and controlling new or ongoing outbreaks. While reduction of prevalence was achieved in all areas, some communities saw significant increases at or above prior baselines around 1 to 3 years later [12]. It is believed that this is due to transient populations where neighboring communities reintroduced scabies infestations back to the treated communities, indicating that wider areas of MDA need to be considered in the future to maintain successful reduction [21].

Good compliance to treatment of the initial case of scabies has been documented from a study in the Northern Territory (with 70% compliance of index cases); however, household contacts of these cases had poor compliance, with only 44% completing treatment [22]. It has also been shown in a trial in Fiji comparing MDA of topical and oral treatments that oral ivermectin had higher efficacy for the control of scabies and impetigo (94% versus 62%), believed to be a consequence of the better ability to observe patients’ compliance with treatment (with 96% of patients using oral ivermectin observed administering treatment compared to only 58% using topical permethrin observed) [8].

The Healthy Skin Program that was implemented by the Cooperative Research Centre for Aboriginal Health (from 2003 to 2009) recognized the importance of not only treating scabies and skin infections in Aboriginal communities, but also focusing on communication and local health promotion to engage and empower locals to help drive scabies control [23]. Over their 6 years of engagement, they demonstrated a reduction in scabies prevalence from 30% to 5% in some areas in the short term [23].

## Future prevention and control in Aboriginal and Torres Strait Islander communities

While diagnosis and reporting of scabies cases in remote Aboriginal and Torres Strait Islander communities have certainly improved in the last 10 to 20 years, there is still great disparity between the Australian states and territories. The Northern Territory has the best data collection for scabies and is able to accurately estimate the prevalence of scabies in remote Aboriginal and Torres Strait Islander communities at between 16% and 35% [11]. In order to accurately prevent and control outbreaks of scabies in remote Aboriginal and Torres Strait Islander Australia, the first step is collecting data about diagnosis and prevalence so that widespread treatment programs can be evaluated and improved.

The Australian National Healthy Skin Guideline has reviewed evidence to provide specific treatment recommendations for Aboriginal and Torres Strait Islander children for topical permethrin (first line) and oral ivermectin (second line), but, most importantly, aggressively treating secondary impetigo infections with systemic antibiotics [11]. The recognition of resistance patterns for treatment of impetigo, and, therefore, the promotion of more aggressive treatment is certainly a step forward; however, the implementation of these guidelines needs to be regularly and rigorously reviewed for efficacy. Although topical permethrin has been shown to be a superior treatment when used as prescribed, compliance issues remain a concern, and so it may be more appropriate for this guideline to be adjusted to include oral ivermectin as the single first line or alongside topical as an option of first-line treatment to increase compliance. Evidence of success in overseas programs should be considered such as MDA in a 2015 Fiji study, which demonstrated that 2 doses of oral ivermectin was more effective than 2 treatments with topical permethrin and reduced prevalence by 94% (from 32.1% to 1.9%) in 12 months [7].

In a multicenter study in France, the safety and efficacy of oral ivermectin against scabies were reviewed in 170 infants weighing less than 15 kgs [24]. They demonstrated an 85% efficacy for scabies clearance and reported no serious adverse effects [24]. As the treatment in infants <15 kgs remains limited in Australia, oral ivermectin should be considered as an option for scabies refractory to topical treatment or those at risk of suffering complications, and, particularly, in remote Aboriginal and Torres Strait Islander communities. Off-label use of oral ivermectin in infants less than 15 kg should be tracked, and research should be encouraged in this area to establish safety and potentially broaden treatment recommendations.

Oral ivermectin is not without side effects, and repeat use has been shown to develop resistance; therefore, new treatments should continue to be sought and investigated [25]. The University of Canberra is currently trialing the efficacy of tea tree oil in treating scabies in children in remote Aboriginal and Torres Strait Islander communities. This is a promising new treatment; however, there is currently limited clinical data to support its widespread use [26]. Caution should also be taken with essential oils as skin treatments as tea tree oil is a known cause of allergic contact dermatitis in some people [27].

The exclusion of oral ivermectin use in infants and pregnant women limits treatment and control in these population groups. Data from countries such as France that allow use in these populations should be closely monitored and reviewed to see if it is appropriate to expand Australian recommendations [20,24].

Addressing social determinants of health of remote Aboriginal and Torres Strait Islander Communities as a whole and employing community consultation to implement widespread health strategies remain challenging objectives, but these are important contributors that need to be addressed to implement effective scabies control programs [11]. There is a great difference in age distribution in the Aboriginal and Torres Strait Islander population compared with non-Aboriginal Australia. A total of 34% of Aboriginal and Torres Strait Islander Australians are under the age of 18 years, compared with just 18% of non-Aboriginal Australians [28]. This younger age profile likely contributes to the high prevalence of scabies among Aboriginal and Torres Strait Islander people as there is a higher risk of transmission in this demographic due to poor capacity of social distancing among children. Controlling scabies outbreaks in remote Aboriginal and Torres Strait Islander communities, particularly among children, remains a significant challenge. Adequate and targeted planning and resources are required to mitigate contributing factors such as overcrowding, unsafe housing, and poor sanitation. Directing efforts toward addressing these challenges now will reduce the risk of outbreaks and improve health outcomes for future generations.
